# Cardiovascular risk and mortality in patients with hyperuricemia treated with febuxostat or allopurinol: a retrospective nation-wide cohort study in Austria 2014–2017

**DOI:** 10.1007/s00296-022-05139-8

**Published:** 2022-05-19

**Authors:** Stefan Weisshaar, Brigitte Litschauer, Berthold Reichardt, Felix Gruber, Stefan Leitner, Sasa Sibinovic, Michael Kossmeier, Michael Wolzt

**Affiliations:** 1 Department of Clinical Pharmacology, Medical University of Vienna, Währinger Gürtel 18-20, 1090 Vienna, Austria; 2 Austrian Health Insurance Fund Burgenland, Eisenstadt, Burgenland Austria; 3 Federation of Social Insurances, Vienna, Austria

**Keywords:** Allopurinol, Cardiovascular disease, Febuxostat, Gout

## Abstract

Patients with hyperuricemia and gout are at an increased risk for cardiovascular (CV) disease. Inhibition of the xanthine oxidase with allopurinol or febuxostat have become the mainstay for urate lowering therapy. However, it has been suggested that febuxostat increases the risk for CV mortality as compared to allopurinol. The aim of this retrospective cohort study was to assess the CV risk among patients with febuxostat or allopurinol therapy. Patients who initiated urate lowering therapy with febuxostat or allopurinol between 2014 and 2017 were selected from the drug reimbursement database of the Austrian health insurances funds. The primary CV endpoint was a composite of angina pectoris, nonfatal myocardial infarction, nonfatal subarachnoid or cerebral hemorrhage, nonfatal ischemic stroke, or death from any cause. In total, 28.068 patients (62.1% male) with a mean age of 71 years were included. 7.767 initiated febuxostat treatment and 20.301 received allopurinol. The incidence rate per 100 patient-years of the composite primary endpoint was 448 (febuxostat) and 356 (allopurinol) with a corresponding adjusted hazard ratio (HR) of 0.58 (95% CI 0.53–0.63) for allopurinol vs. febuxostat initiators. Similar HR were found for secondary endpoints including all-cause mortality [0.61 (95% CI 0.55–0.68)] and separate analyses of cardiac events [0.48 (95% CI 0.38–0.61)] and ischemic stroke [0.47 (95% CI 0.36–0.61)]. Data from this Austrian population-based study suggests that febuxostat initiators are at an increased risk for nonfatal CV events or death from any cause as compared to those with allopurinol. This is consistent with CV concerns of other trials, which limited the broad therapeutic use of febuxostat.

## Introduction

Gout is a common chronic inflammatory condition, which is associated with hyperuricemia resulting from a perturbed uric acid metabolism. Patients with gouty arthritis suffer from acute or episodic painful flares due to deposition of monosodium urate crystals in peripheral joints [1–3]. Both the prevalence and incidence of gout are increasing and epidemiologic data suggest numerous risk factors such as the aging of the population, lifestyle/alimentation changes, obesity, diabetes, or the use of diuretic medicines [4]. Further, patients with gout are considered to be at a higher risk for cardiovascular (CV) disease, chronic kidney disease, or premature mortality as compared to those without gout [4–6].

Pharmacological treatment strategies for hyperuricemia include, among others, administration of xanthine oxidase inhibitors (XOI) such as allopurinol or febuxostat. Both medicines are approved for lowering serum uric acid concentrations to prevent future flares [7]. Allopurinol and febuxostat improve hyperuricemia by impeding the formation of xanthine from hypoxanthine and of uric acid from xanthine, respectively [8]. Febuxostat is more potent than allopurinol and does not require dose adjustment in patients with mild to moderate impaired renal function [9–11]. However, data from clinical trials suggest that the rate of CV events in patients treated with febuxostat is higher than in patients receiving allopurinol or placebo [10, 12]. Further, the Cardiovascular Safety of Febuxostat and Allopurinol in Patients with Gout and Cardiovascular Morbidities (CARES) trial has shown that febuxostat increased the risk for all-cause and CV mortality as compared to patients receiving allopurinol [13]. The US Food and Drug Administration (FDA) concluded accordingly that there is an increased risk for death with febuxostat and limited its therapeutic use [14]. In contrast, data from a large population-based trial in Korea did not show any differences on mortality or CV events in febuxostat-treated gout patients as compared to those receiving allopurinol [15].

The aim of the present retrospective epidemiological nation-wide study was to assess the effect of allopurinol or febuxostat treatment among Austrian patients with hyperuricemic conditions on CV event risk and mortality. CV events were defined as hospitalization due to angina pectoris, acute myocardial infarction (AMI), ischemic stroke, or subarachnoid or cerebral hemorrhage. Analysis was performed from an observational period of 4 years by evaluation of data derived from the Federation of Social Insurances.

## Methods

The protocol of this retrospective study was approved by the Ethics Committee of the Medical University of Vienna, Austria (EK 2104/2018). The study conforms to the principles outlined in the Declaration of Helsinki including current revisions. The study period ranged from January 1, 2014 to December 31, 2017.

### Data source

Austria´s health insurance system provides health-care benefits for residents who are assigned to one of the several health insurances funds according to their current or former employment, and province of residence. Information of medical treatment covered by the health insurance funds is recorded in the respective databases, comprises about 97% of the Austrian population and is reported to the Federation of Social Insurances. These data include, among others, demographics, medical discharge diagnoses of hospital stays using the 10th revision of the International Statistical Classification of Diseases and Related Health Problems (ICD 10) coding system and prescriptions of medicines reimbursed by the Austrian Social Insurances (i.e., all medicines with either costs that exceed the respective prescription charge [EUR 5.40—EUR 5.85 for 2014–2017] or which are prescribed to patients who are exempt of paying the prescription charge). For this retrospective cohort study, we utilized longitudinal pooled data from the Federation of Social Insurances for the period of January 1, 2014 to December 31, 2017. Data were pseudonymized to preserve patients’ privacy. Data storage and handling were in agreement with the applicable data protection laws.

### Study cohort

Eligible patients were 18 years or older and initiated a urate-lowering therapy with febuxostat or allopurinol between January 1, 2014 and December 31, 2017. XOI initiators were defined as individuals who did not fill any prescription of febuxostat (ATC code: M04AA03) or allopurinol (ATC code: M04AA01) prior to 2014. Patients were required to receive at least six continuous dispensings of the respective XOI within the observational period. This inclusion criterion was introduced to establish a possible causal relationship between XOI intake and adverse cardiovascular events [16]. Individuals who received prescriptions for both XOI medicines during the period of interest or had a history of malignancy (ICD code: CXX) were excluded.

### Outcome definition

The primary outcome parameter was defined as a composite end point of the first occurrence of hospitalization due to angina pectoris (ICD-10 code: I20), nonfatal myocardial infarction (I21), nonfatal subarachnoid (I60) or cerebral hemorrhage (I61), nonfatal ischemic stroke (I63), or death from any cause during XOI intake or within ≤ 180 days after discontinuation of the XOI medicines. Secondary end points included separate analyses of cardiac events (composite of I20 and I21), intracranial bleeds (composite of I60 and I61), ischemic stroke (I63), and all-cause mortality.

### Covariate assessment

Variables that were potentially associated with the baseline risk regarding CV outcome parameters of interest and that have been recorded in the Federation of Social Insurances database were assessed at time of treatment initiation. These variables included demographics (age, sex) and medicines that are commonly prescribed for treatment of CV risk factors. The medicines served as surrogates for co-morbidities and included antihypertensive medicines (beta blocker [ATC code: C07], angiotensin-converting enzyme inhibitors (ACE-I) and angiotensin receptor blockers (ARB) [ATC code: C09]), antidiabetics (ATC code: A10), antiplatelet medicines (B01AC) and lipid-lowering agents (ATC code: C10). These variables were used as control variables to account for potential differences between the febuxostat and allopurinol cohort already present at treatment initiation. Other risk factors, such as smoking behavior, or laboratory or other diagnostic tests were not included as these data are not available in the Federation of Social Insurances database.

### Statistical analysis

Statistical evaluations were performed with R (Version 4.1.0). Continuous and categorial variables are described by means, standard deviations (SD), absolute frequencies and/or percentages. The survival time distribution was estimated using the Kaplan–Meier estimator. The median follow-up time was estimated by the inverse Kaplan–Meier method (i.e., by inverting the event indicator). Cox proportional-hazard regression models were applied to estimate the association between febuxostat or allopurinol use and the time to the first occurrence of the composite primary or secondary end point events. Cox regression models were controlled for sex, age, and concomitant medication at baseline (treatment initiation). Adjusted hazard ratios (HR) with 95% confidence intervals (CI) were computed for primary and secondary outcome parameter associated with febuxostat or allopurinol use. The follow-up time started the day after the index date (i.e., first dispensing of febuxostat or allopurinol) and continued until XOI medicine discontinuation + 180 days, occurrence of an end point event, or end of study follow-up. Patients without a primary end point event during the 4 year observational period had their data censored on December 31, 2017 (database lock). If febuxostat or allopurinol treatment was discontinued during the study period, patients were censored at 6 months (i.e., 180 days) after the last dispensing of the XOI medicines.

## Results

### Cohort selection and patients’ characteristics

**Fig. 1:**
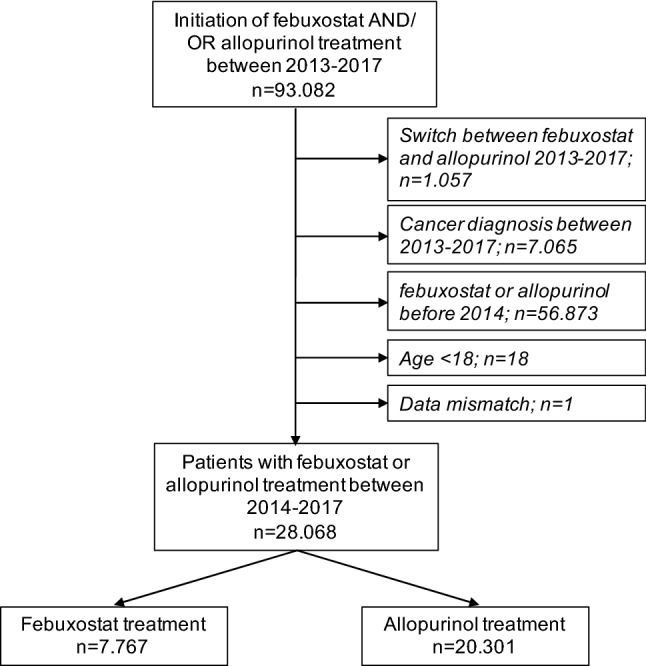
Study cohort selection

Figure 1 shows the study cohort selection process. In total, 28,068 patients who received XOI treatment and met the inclusion criteria were identified. 7767 subjects were febuxostat initiators and 20,301 were treated with allopurinol, respectively.

Table 1 summarizes demographics and concomitant medication of the study cohorts. There were more male patients receiving febuxostat as compared to allopurinol. Allopurinol initiators had higher numbers of co-medication at baseline (treatment initiation) than febuxostat initiators. The median (95% confidence interval) follow-up time was 640 (628–654) days in the febuxostat cohort and 907 (901–914) days for allopurinol initiators.

**Table 1:** Patient characteristics

	Febuxostat initiators	Allopurinol initiators
	(*n* = 7.767)	(*n* = 20.301)
Demographics		
Age, years	69 (13)	71 (12)
Male sex, %	74.1	57.5
No. of dispensings	15 (9)	11 (6)
Concomitant medication, %		
Lipid-lowering agents	51.7	58.2
Antidiabetics	25.8	34.0
Antiplatelet medicines	24.7	36.3
Antihypertensive medicines		
Beta blocker	50.0	59.4
ACE-I or ARB	75.5	79.7

Data are mean (SD)

*ACE-I* angiotensin-converting enzyme inhibitors; *ARB* angiotensin receptor blockers

### Risk of cardiovascular disease and mortality in febuxostat vs. allopurinol initiators

**Fig. 2:**
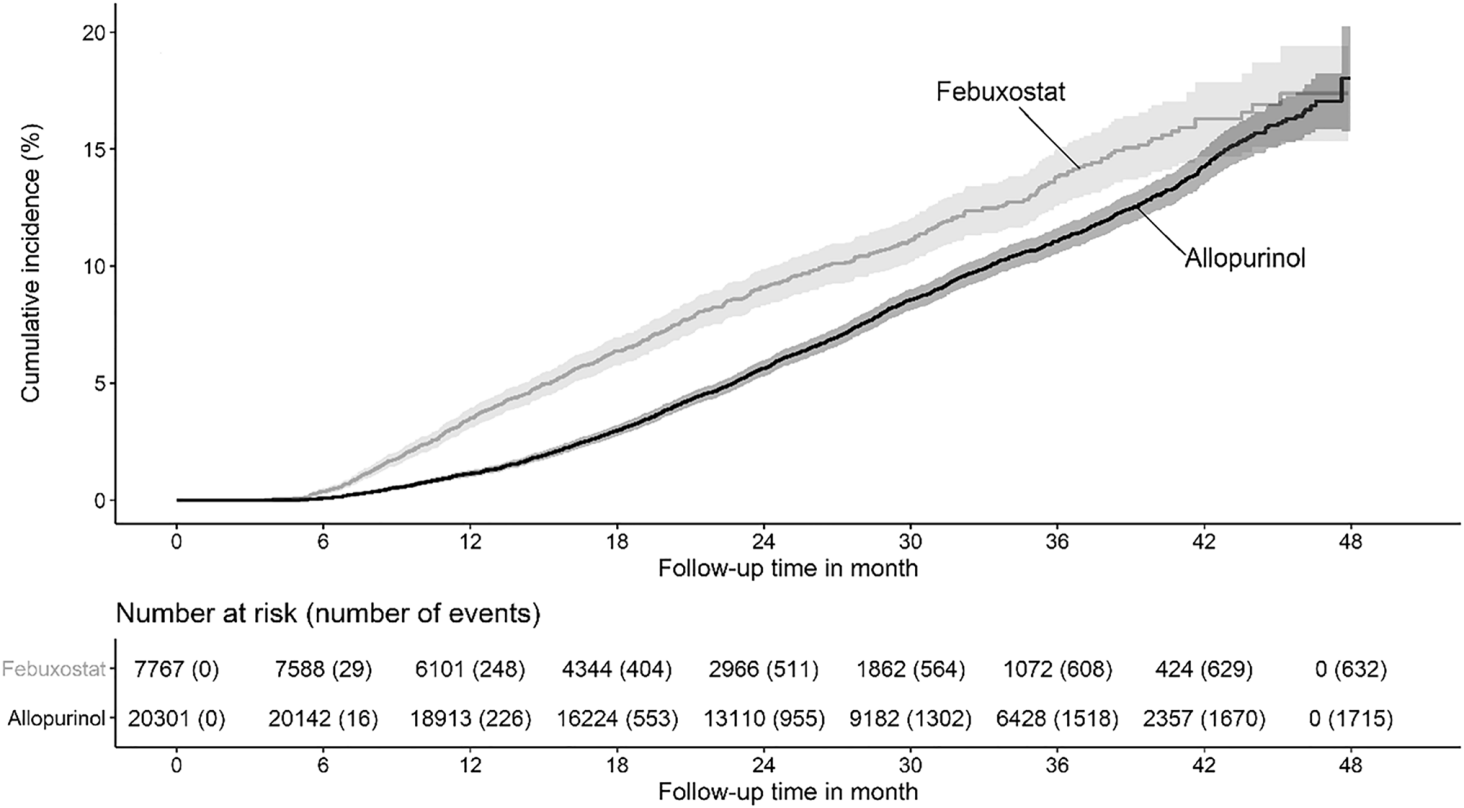
Kaplan–Meier estimates of the time to the first occurrence of the composite primary end point (hospitalization due to angina pectoris, nonfatal myocardial infarction, nonfatal subarachnoid or cerebral hemorrhage, nonfatal ischemic stroke, or death from any cause)

Kaplan–Meier curves of survival probability for the composite cardiovascular end point are presented in Fig. 2. Table 2 summarizes incidence rates of primary and secondary end points, which were all higher—except for intracranial hemorrhage—in the febuxostat cohort as compared to allopurinol. Febuxostat treatment was associated with a greater risk for composite cardiovascular events or death during treatment with febuxostat as compared to allopurinol (adjusted HR: 0.58; 95% CI 0.53–0.63; *P* < 0.001). Likewise, a similar pattern was observed for cardiac events (myocardial infarction or angina pectoris), stroke or death, respectively (Table 2). The risk for intracranial hemorrhage was also numerically lower in the allopurinol cohort, but did not significantly differ between study cohorts.

**Table 2:** Outcome parameter

	Febuxostat initiators		Allopurinol initiators		
	*n* = 7.767		*n* = 20.301		
	Events	IR	Events	IR	Adjusted HR (95% CI)
Composite end point	632	4.48	1715	3.56	0.58 (0.53–0.63)*
AP or MI	107	0.76	239	0.50	0.48 (0.38–0.61)*
Stroke	78	0.55	176	0.37	0.47 (0.36–0.61)*
IH	8	0.06	32	0.07	0.75 (0.34–1.66)
Death	439	3.11	1268	2.63	0.61 (0.55–0.68)*

*IR* incidence rate per 100 patient-years; *AP* angina pectoris; *MI* myocardial infarction; *IH* intracranial hemorrhage

*P < 0.001

Female patients were at lower risk for the combined primary end point (adjusted HR: 0.78; 95% CI 0.71–0.85; *P* < 0.001), death (adjusted HR: 0.78; 95% CI 0.71–0.86; *P* < 0.001), or myocardial infarction or angina pectoris (adjusted HR: 0.54; 95% CI 0.42–0.69; *P* < 0.001). The risk for other secondary end points did not show an association with sex.

Treatment with ACE-I/ARB or lipid-lowering agents had protective effects on the composite primary end point or mortality. Beta-blockers, antidiabetic, or antiplatelet medicines were associated with an increased risk for occurrence of these end points. ACE-I/ARB, lipid-lowering medicines or antiplatelet drugs administration were associated with an increased risk of cardiac events (myocardial infarction or angina pectoris), while other concomitant medicines had no impact. Hospitalization for stroke or intracranial hemorrhage was not affected by the concomitant medication under study.

## Discussion

Our findings show that treatment with febuxostat substantially increased the risk for death from any cause and non-fatal CV events except for intracranial hemorrhage. These findings are partly consistent with the results of the CARES trial, which led to warnings for the use of febuxostat in some jurisdictions [14]. CARES had higher numbers in both all-cause mortality and CV death in patients with febuxostat treatment as compared to allopurinol. Non-fatal CV events were similar between allopurinol and febuxostat in the CARES population. This is at variance with our results and may be due to a more diverse study population in the present study than in the CARES cohort, which required a history of CV disease as inclusion criteria [13]. Likewise, it cannot be excluded that subjects of our study population had insignificant symptoms of CV disease (e.g., mild angina pectoris) at baseline as in our database outpatient visits (e.g., at the cardiologist) are not recorded with diagnoses. Although CARES was conducted in a randomized controlled fashion, a selection bias cannot be excluded due to the high numbers of premature study drug discontinuation (57%) and of patients lost to follow-up (45%) [17].

Other data of retrospective population studies [15, 18] did not show any significant difference in CV disease or mortality end points in febuxostat vs. allopurinol initiators. Notably, subjects in a Korean trial [15] were significantly younger than in our study cohort (60 vs. 71 years), which may have affected outcomes. We observed a more than two-fold higher incidence rate in the composite end point in both allopurinol and febuxostat initiators suggesting that included patients in our study cohorts were sicker. Likewise, this observation may be age-related but also due to differences in socioeconomic variables in Europe vs. Asia. However, co-variate assessment was limited since medical history data, results from laboratory analysis or secondary medical discharge diagnoses were not available for our analysis. Thus, data provided from hospitals to the Austrian insurance institutions may be incomplete and the most common prescribed medicines in Austria [19] were used as surrogates for co-morbidities.

Proportions of concomitant medication in allopurinol initiators were higher than in patients with febuxostat suggesting that patients treated with allopurinol had a higher number of co-morbidities. This finding is supported by the fact that costs for allopurinol are below Austria´s prescription charge and therefore primarily allopurinol dispensings to prescription fee exempted patients are recorded in the databases of insurance institutions. This population group has usually a lower income and it is generally known that a lower socioeconomic status is associated with a higher number of co-morbidities [20]. Although concomitant medication was statistically adjusted for in our analysis, a selection bias particularly in the allopurinol study cohort cannot be excluded and it is likely that patients with a lower socioeconomic status are overrepresented in allopurinol initiators.

An immortal time bias, which may have over- or underestimated the event rates in different study groups, cannot be excluded [21]. Although data from 2013 to 2017 was available, we introduced a “cooling-off phase” of one year and excluded subjects with XOI treatment before 2014 to provide more robust statistics. Both the duration of allopurinol/febuxostat intake or if patients had received any XOI treatment earlier would have remained unclear as numbers of prescriptions filled in 2012 or earlier were not available. Further, only patients were included who filled at least six continuous XOI prescriptions to provide an appropriate observational period of drug exposure.

Febuxostat has been shown to be a more potent urate lowering medicine than allopurinol [7] and there is evidence that an excessive lowering of serum urate levels (below 4–5 mg/dL) may increase the risk for CV events through mitigation of uric acid´s beneficial antioxidant effects [22–24]. It cannot be excluded that uric acid levels were excessively reduced by febuxostat resulting in a higher CV risk or mortality in febuxostat initiators, but this is unclear in the absence of serum urate levels. In CARES a higher proportion of patients achieved lower serum urate levels (< 5 mg/dl) during febuxostat treatment as compared to allopurinol [13]. Likewise, it is also possible that patients in our study population were inadequately treated and under-dosed with febuxostat or allopurinol. Previous data suggest that adherence to urate-lowering therapy is poor and gout treatment suboptimal [25].

By using an active comparator (allopurinol vs. febuxostat) rather than a placebo-controlled design for this retrospective study and including patients without any XOI treatment at least for one year by using a “cooling-off phase”, potential confounding is minimized [26]. Another strength of the present study, which may increase generalizability, is that cardiovascular safety of different XOI treatments was assessed in a representative European elderly population and not only in those with CV disease at baseline like in CARES [13].

However, our retrospective study has several limitations. The ICD-10 coding of hospital discharge diagnoses was used to identify eligibly patients with CV events or mortality and a classification bias cannot be excluded. Further, analyses of outcome parameters are limited to XOI initiators who have been discharged following hospitalization and patients from ambulatory care are not represented in the study cohorts. Data from Austria´s insurance institutions do not provide information on cause-specific mortality, medical history or laboratory analyses. Additional confounding may arise by using ATC codes for disease-specific medicines as surrogates for co-morbidities due to limited information (e.g., incomplete coding for secondary discharge diagnoses). Information of prescribed medicines is also limited to those which are reimbursed by the health care provider and the use of over-the-counter medicines remain unknown. In particular, information of prescription free nonsteroidal anti-inflammatory drugs, which have been shown to be associated with an increased CV risk [27], is not available. Furthermore, it is not possible to conclude drug adherence with these data.

In conclusion, initiation of XOI treatment with febuxostat was associated with an increased risk for CV adverse events or all-cause mortality as compared to patients receiving allopurinol as assessed from retrospective data of hospital discharge diagnoses in Austria. This is consistent with concerns of other trials and supports cautious use of febuxostat in patients with increased CV risk.

## Data Availability

The datasets generated and analyzed are available from the corresponding author upon reasonable request.
